# Abstract of the Proceedings of the Buffalo Medical Association

**Published:** 1876-06

**Authors:** E. N. Brush

**Affiliations:** Secretary


ART. IV.—*Abstract of the Proceedings of the Buffalo Medical Association.* Tuesday Evening, April 4th, 1876. E. N. BRUSH, M. D., Secretary.

The President, Dr. Gould, in the Chair.

Present—Drs. White, Rochester, Miner, Phelps, O'Brien, Little, Diehl, Brecht, Fowler, Howe, Bielby, Barnes, Coxe, Briggs, Samo, Macniel, Mynter, Van Peyma, Gay, Trowbridge, Hebenstreit, Hopkins, Bartlett and W. W. Miner. By invitation, Drs. Baker, Shannon and Elliott.

The minutes of the Secretary were read and approved.

The annual report of the Treasurer, Dr. Fowler, was received, and referred to an auditing committee consisting of Drs. Phelps, Gay and Barnes, who subsequently reported the accounts correct.

The Sanitary Committee appointed at the February meeting, reported through Dr. Barnes. On motion of Dr. Rochester, the report was placed on file.

The application for membership of Drs. S. S. Greene, S. G. Dorr, W. C. Earl and W. H. Slacer were taken from the table, and they were unanimously elected members.

The President appointed as Essayist for the May meeting, Dr. E. C. Coxe.

DR. HOWE then presented a post mortem and a clinical specimen of that form of intra-ocular tumor known as glioma.

The first was from a child five years old, whose parents noticed, in the fall of '73, that the pupil of the right eye was enlarged and of a yellowish color. The vision was then lost. This patient was seen in consultation with Dr. Rochester, on the tenth of April, '75. At that time the eye was prominent, the sclerotic being much distended. The cornea was clear, and behind, instead of an anterior chamber, the iris and lens were pressed directly against it. The pupil was widely dilated, and through it was seen a bright yellowish reflection. The patient suffered continual pain and was much emaciated. The diagnosis being agreed upon, the eye was enucleated, and the growth found to occupy its posterior half. Its malignant character as a glioma retinæ was shown by the microscope. In spite of its removal however, the other eye became affected, the patient gradually sank, and died on the twelfth of

June following. A post mortem examination of the remaining eye shows a condition similar to that already described in the right.

The other case is also that of a little girl, of six years. The tumor was first noticed at the outer margin of the iris, and now, by looking around the edge of the pupil, it can be readily seen without the aid of ophthalmoscope or any other instrument.

(The question of differential diagnosis between glioma and other tumors was then discussed.)

These growths are of interest.

1st. From their extreme infrequency.

2d. From the comparative ease with which they can be recognized in their more advanced stages by the yellowish reflex.

3d. From their fatal termination, which makes the removal of the eye advisable, simply for the sake of prolonging life, and hardly with the expectation of saving it.

Dr. Mynter read the report of a second case of aspiration of the pleural cavity for effusion of acute pleuritis. (See page 383 May No.

Dr. Rochester said that sufficient time had not elapsed to say whether the effusion would return or not, and therefore the case could not be accepted as a cure of pleuritis by thoracentisis. He referred to a case which had come under his observation some time since. The patient, a gentleman, had had pleuritic effusion three times in two years; twice on the left side, once on the right. The recovery was perfect, but it was not promoted by the ordinary treatment, the patient was put upon tonics, cod liver oil, iron, etc., under which, improvement was marked and rapid.

Dr. S. S. Greene said that he was very much pleased to hear Dr. Mynter's paper. That he was the gentleman who had advised Dr. Mynter's patient to defer having the operation performed. He had advised the employment of other, and less radical means for relief, saying to the patient that the operation, in the condition which he saw, was not necessary, as he was suffering from no dyspnœa or other unpleasant symptoms. The man came to him for treatment, having the appearance of one who was in the habit of drinking to excess, he moreover understood that the patient had recently been under treatment for general anasarca, from which he had not wholly recovered. If the patient had been in danger from too great pressure upon the lung, or had been suffering from great dyspnœa he should have advised the employment of the aspirator, but unless these conditions were present he should prefer to use other means.

Dr. Barnes said that he had occasion to assist a gentleman, a few days since, in tapping the chest on the fifth day, of acute pleuritis. The operation, though affording relief at the time, was followed by a rapid return of the effusion.

Dr. Miner wished to call attention to the fact, that these cases even in the most extreme instances, would recover. He cited the case of a boy, aged about fourteen, who came under his care for pleuritic effusion. When seen the effusion had become purulent and had pointed in the side. The effusion was drawn off by an ordinary lancet, the incision being made in the most dependent portion of the swelling. The recovery of the patient was rapid. He had seen, in an extended experience, several extreme cases of this character, and had been surprised frequently, at their favorable termination. He did not think that in the early stages of pleuritic effusion, the danger to the patient or the prospect of hastening his recovery were sufficiently well established to warrant the use of the aspirator.

Dr. Rochester moved that as the hour was late the subject be laid upon the table until the next meeting. Carried.

Dr. White said that he had a case to lay before the Association, which was to him full of interest. The case was one of non-puerperal hemorrhage after the menopause, there were no indications of malignant disease. The patient was a widow aged forty-nine, who had never borne children. Two or three years ago he had treated the patient for slight hemorrhage accompanied by a small amount of ulceration of the os. Was sent for again last summer to see the patient, but through a mistake of the messenger did not find her, and did not see her again until February 6th. She was found very much exsanguined from repeated hemorrhages which had occurred for some months. The profuse hemorrhage then present was controlled by the tampon. The hemorrhage did not again recur, but was succeeded by a profused watery or serous discharge, such as would be expected from polypoid growths. On the 15th and 16th of March, teuts were introduced, the os dilated and the uterus measured, its cavity being found to be three inches in length. Dr. Coxe anæsthetized the patient, and with the curette of Récamier, Dr. White removed from the cavity of the uterus what were apparently small polypoid growths springing from the mucous membrane. On that evening and the next day the patient was in a comfortable condition, but sank and died in a day or two after. There were none of the ordinary symptoms of peritonitis nor did the patient suffer from much pain. A *post mortem* examination was made by Drs. Folwell, Coxe and Fowler, and the uterus, which they had removed, was presented for inspection. The body of the uterus was found to be soft and putty like and had a ragged perforation at the fundus. The uterus seems to have undergone a process of fatty degeneration. The neck and os were comparatively free from disease, and were firm and elastic while the body and fundus were almost as soft as tallow, as could be seen by an examination of the specimen. A minute detail of the case after the operation was furnished by Dr. Coxe, who was in attendance until the termination of the case.

The only disease reported prevailing in the city to any general extent was a severe epidemic of influenza.

Drs. White, Briggs and Van Peyma were appointed a nominating committee to select officers for the ensuing year. They brought in the following ticket, which was unanimously elected.

*For President,* Dr. C. C. Wyckoff,
*For Vice-President,* Dr. E. R. Barnes,
*For Secretary,* Dr. E. N. Brush.
*For Treasurer,* Dr. Joseph Fowler,
*For Librarian,* Dr. J. B. Samo.

On motion the Association adjourned.

